# Population Structure and Genetic Diversity of Yunling Cattle Determined by Whole-Genome Resequencing

**DOI:** 10.3390/genes14122141

**Published:** 2023-11-27

**Authors:** Jian Chen, Lilian Zhang, Lutao Gao, Zaichao Wei, Dong Dang, Linnan Yang

**Affiliations:** 1 State Key Laboratory of Agricultural Big Data, Academy of Big Data, Yunnan Agricultural University, Kunming 655508, China2021110014@stu.ynau.edu.cn (Z.W.);; 2 School of Data Science, Baoshan University, Baoshan 678000, China

**Keywords:** Yunling cattle, genetic diversity, population structure, whole-genome resequencing

## Abstract

The Yunling cattle breed, a three-breed crossbreed, which comprises 50% Brahman cattle, 25% Murray Grey cattle and 25% Yunnan Yellow cattle, has several advantageous traits, including rapid growth, superior meat quality, ability to improve tolerance in hot and humid climates, tick resistance and rough feed. It can be rightfully stated that Yunling cattle serve as vital genetic repositories of the local Yunnan cattle. Gaining insights into the genetic information of Yunling cattle plays a significant role in the formulation of sound breeding strategies for this breed, safeguarding genetic resources and mitigating the risks associated with inbreeding depression. In this study, we constructed the Yunling cattle standard reference genome and aligned the whole genomes of 129 Yunling cattle individuals to the constructed reference genome to estimate the current genetic status of Yunling cattle in Yunnan Province, China. The average alignment rate and the average percentage of properly paired are both 99.72%. The average nucleotide diversity in Yunling cattle is 0.000166, which indicates a lower level of diversity. Population structure analysis classified Yunling cattle into two subgroups. Inbreeding analysis revealed that inbreeding events did occur in the Yunling cattle, which may have contributed to the low genetic diversity observed. This study presents a comprehensive assessment of the genetic structure and diversity among the Yunling cattle and provides a theoretical foundation for the preservation and exploitation of these precious germplasm resources.

## 1. Introduction

Yunling cattle is a breed developed by Yunnan Academy of Grassland and Animal Science in China. It is a three-breed crossbreed, which comprises 50% Brahman cattle, 25% Murray Grey cattle and 25% Yunnan Yellow cattle, and it has various benefits, such as rapid growth, superior meat quality, improved tolerance to hot and humid climates, tick resistance and tolerance to crude feeds. The transverse fixup has reached the fifth generation. It is primarily distributed across numerous regions within Yunnan Province and minimally dispersed throughout adjacent provinces [1,2]. Livestock and poultry germplasm resources are strategic resources of the country, and the preservation of local breeds is crucial to the implementation of the seed industry revitalization strategy. With these advantageous traits, the Yunling cattle breed has emerged as a significant resource for beef production and cattle breeding in China.

Genetic diversity is the cornerstone of biological adaptation to the environment and evolution and serves as a vital reference for assessing the status of germplasm resources [3]. The richer the genetic diversity within a population, the stronger its ability to adapt to environmental alterations [4]. Assessing the genetic diversity in a population aids in comprehending its present status and future prospects pertaining to germplasm resources, playing a crucial role in the safeguarding, growth and usage of livestock and poultry germplasm resources [5]. Therefore, the assessment of the genetic characteristics of Yunling cattle is of great necessity for the construction of a rational breeding strategy for Yunling cattle and the design of a local conservation programme for Yunnan cattle [2]. The genetic diversity in Yunling cattle has been systematically investigated using various methods, including karyotypic analysis [6], microsatellite DNA markers [1] and the study of Y-chromosome polymorphisms, including Y-SNPs and Y-STRs [7]. Here, we provide supplementary insights into the genetic diversity in the Yunling breed from a comprehensive analysis of whole-genome DNA. According to previous research, DNA genetic diversity has revealed the global and local components of ancestry [8]. However, the current genetic status of Yunling cattle remains uncertain, particularly at the genome-wide level.

In this study, we used 129 Yunling genomes and compared them with the standard genome of Yunling cattle (BioProject accession number PRJNA978937) and assessed genetic status through calculating nucleotide diversity and heterozygosity indexes. Subsequently, we evaluated the population structure of Yunling cattle through ADMIXTURE, principal component analysis (PCA) and construction of a neighbour-joining tree at the genomic level. Then, the inbreeding status of the Yunling cattle population was analysed by calculating the inbreeding coefficient and fixation index. Our results will aid in the comprehension of the population structure and genetic characteristics of Yunling cattle and establish a base for breeding Yunling cattle and maintaining the genetic diversity in Yunling cattle.

## 2. Materials and Methods

### 2.1. Samples and Sequencing

A set of biological tissues were sampled from a four-year-old male Yunling cattle reared at Chuxiong JinDa Farm of Chuxiong City in Yunnan Province and quickly frozen in liquid nitrogen. Among them, heart tissues were utilised for DNA sequencing in order to assemble the genome. Genomic DNA from heart tissue was extracted using the standard phenol-chloroform [9] extraction method for DNA sequencing library construction. The integrity of the genomic DNA molecules was checked using agarose gel electrophoresis. We used the BGISEQ DNBSEQ-T7 platform for short sequencing (bp) to obtain 161.89 GB raw data (64X coverage of the estimated genome size), and the PacBio Sequel II platform (CCS mode) for long sequencing to obtain 61.81 GB raw data for genome assembly. The sequencing work has performed at GrandOmics Biosciences Co., Ltd. (Wuhan, China). In this study, raw sequencing information used to assess Yunling cattle genetic diversity comes from NCBI with the BioProject accession number PRJNA555741 (Supplementary Table S1). In project PRJNA555741, all samples were obtained from Xiaoshao Farm of the Yunnan Academy of Grassland and Animal Science, Kunming, Yunnan, China. During the sampling procedure, animals were chosen on the basis of pedigree information to minimise relatedness between individuals [2].

### 2.2. Assemble and Annotation

We assessed the genome size and heterozygosity by 27-mer analysis with short paired-end reads by using KMC [10] and GenomeScope program [11]. Initial assembly was performed using the HiFiasm program with HiFi long reads, followed by four rounds of correction using Nextpolish with short reads and default settings in order to imporve assembly accuracy. HiC-Pro [12] and fastp were implemented for the filtration of low-quality Hi-C data and paired-end reads, respectively. Clean paired-end reads were subsequently aligned to the assembly utilizing bowtie2 [13] in order to acquire unique paired-end reads. HiC-Pro was used to identify the validation paired-end reads from the unique mapped paired-end reads of interaction paired-end reads while filtering out invalid read pairs. The scaffolds were further clustered, ordered and oriented onto chromosomes by LACHESIS [14]. Finally, Juicebox [15] was utilised for manual correction of large-scale inversions and translocations, resulting in the generation of the ultimate pseudochromosomes.

Then, we performed gene function annotation and annotation of non-coding RNAs (ncRNAs) on the genome. For gene function annotation, we used the default parameters of the InterProScan [16] program to identify putative domains and GO (Gene Ontology) terms of genes, and used Blastp to compare the EvidenceModeler-integrated protein sequences with well-known databases, SwissProt, NR (Non-Reduntant Protein Database), KEGG (Kyoto Encyclopedia of Genes and Genomes), KOG (Eukaryotic Orthologous Groups of protein), with an E value cutoff of $1\times 10^{-5}$ and the results with the hit with lowest E value was retained. By combining the comparison outcomes of the aforementioned databases, the total count of annotated genes amounts to 19,172, which corresponds to 92.80% of the predicted protein-coding genes. For ncRNAs annotation, two strategies were used: searching against a database and prediction with a model. Transfer RNAs (tRNAs) were predicted using tRNAscan-SE [17] with eukaryote parameters. MicroRNA, rRNA, small nuclear RNA and small nucleolar RNA were detected using Infernal cmscan to search the Rfam [18] database. The rRNAs and their subunits were predicted by RNAmmer [19].

### 2.3. Reads Mapping and SNPs Calling

In order to obtain high-quality reads, we utilised fastp for the filtration of low-quality reads (-n 0 -f 5 -F 5 -t 5 -T 5 -q 20). Pair-end sequence reads were filtered and aligned to the Yunling cattle reference genome (BioProject accession number PRJNA978937) using BWA-mem [20] with default parameters. To remove potential duplicate reads, we used the “MarkDuplicates” modules of the Genome Amalysis Toolkit 4.4.0.0 (GATK) [21]. The raw SNPs were identified by utilising GATK’s tools, including “HaplotypeCaller”, “ GenomicsDBImport”, “ GenotypeGVCFs” and “SelectVariants”. To ensure high-quality SNPs, the “VariantFiltration” module was utilised to filter the raw SNPs based on the GATK-recommended hard filtering (QD < 2.0, FS > 60.0, MQ < 40.0, MQRankSum < −12.5, ReadPosRankSum < −8.0 and SOR > 3.0) and average sequencing depth ((for all individuals) <1/3X and >3X). Based on the assembly and annotation files [22] we assembled, the retrieve_seq_from_fasta.pl module of ANNOVAR was used to generate a transcribed FASTA file for the database, and then the annotate_variation.pl module of ANNOVAR was used to perform functional annotation for each SNP [23].

### 2.4. Linkage Disequilibrium and Genetic Diversity Analysis

We used PopLDdecay [24] to calculate the decay of linkage disequilibrium (LD) (-MaxDist 300). We utilised PLINK 1.90 [25] to filter out SNPs, removed SNPs (--geno 0.02) and individuals (--mind0.02) with high deletion rates. Then, VCFTOOLS 0.1.17 [26] was used to filter SNPS by removing indel (--remove-indels) and filtering lower MAF (--maf 0.05 --max-maf 0.95). After that, VCFTOOLS was used for nucleotide diversity (Pi) estimation with the parameters ‘--window-pi 10000 --window-pi-step 5000’, and PLINK 1.90 was used for calculating $H_{O}$ and $H_{E}$ with default settings.

### 2.5. Population Structure, Principal Component Analysis and Phylogenetic Tree Analysis

The population structure of Yunling cattle was studied with the use of ADMIXTURE 1.3.0 software [27], with the assumed assumption that K ancestry was between 2 and 19. We calculated the genetic distance matrix between each individual using PLINK tool. Based on this matrix, we performed PCA analysis using Genome-wide Complex Trait Analysis 1.94.1 (GCTA) [28] and constructed the phylogenetic tree using the neighbour-joining method in MEGA v7.0 [29].

### 2.6. Inbreeding Analysis and Subgroup Analysis

To assess the extent of inbreeding in the Yunling cattle population, we used PLINK with the parameter ‘--het’. Fixation indexes were calculated to estimate the degree of inbreeding in Yunling cattle using VCFTOOLS with the command ‘--fst-window-size 20000 --fst-window-step 5000’. Based on the result of ADMIXTURE, we also calculated $H_{E}$/$H_{O}$ and $F_{HOM}$ for each subgroup separately to evaluate the inbreeding status of each subpopulation.

## 3. Results

### 3.1. Diversity in Single-Nucleotide Polymorphisms, Genetic Diversity

**Figure 1:**
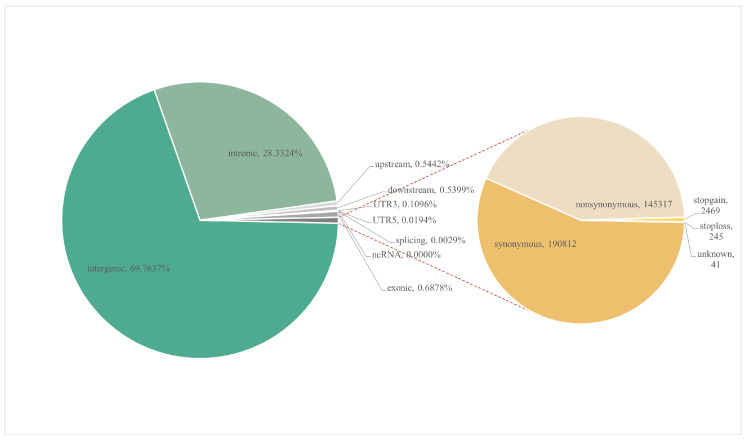
Functional classification of the detected SNPs. The pie chart on the left shows the composition of SNPs with the percentage of each of the following components labelled: intergenic, intronic, exonic, upstream, downstream, UTR3, UTR5, splicing and ncRNA. The pie chart on the right displays the distribution of exons and labels the respective number of each type, including synonymous, nonsynonymous, stopgain, stoploss and unknown variants.

**Figure 2:**
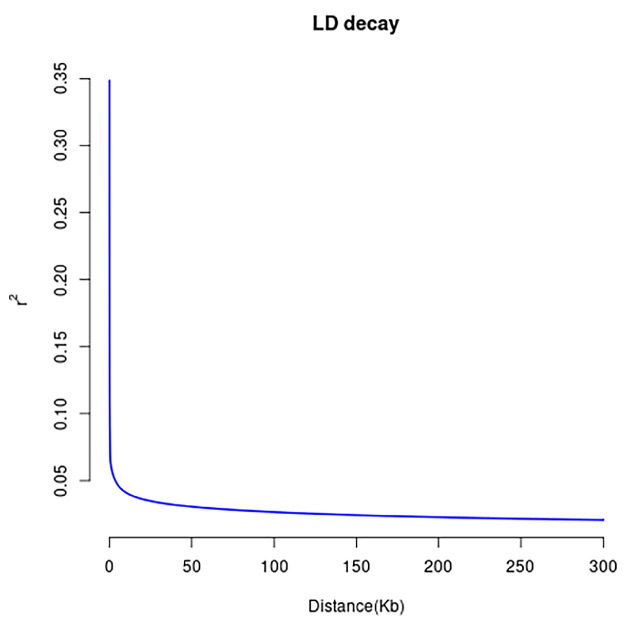
Genome-wide average LD decay estimated for Yunling cattle. The vertical axis denotes the LD coefficient $r^{2}$ and the horizontal axis denotes the distance between the genes.

More than 15 billion clean reads were aligned to the Yunling cattle reference genome sequence (BioProject accession number PRJNA978937), with an average alignment rate of 99.72% and an average percentage of properly paired of 99.72% (Supplementary Table S2). More than 49 million SNPs were identified; the density plots for filtered SNP quality are shown in Figure S1. The majority of the SNPs were detected in intergenic regions, which accounted for 69.8% of the total. Meanwhile, the remaining SNPs located in the open reading frame upstream and downstream each accounted for 0.5% of the total, while those located in introns and exons accounted for 28.3% and 0.7%, respectively. In exons, there are 145,317 non-synonymous CNVs and 190,812 synonymous CNVs (Figure 1). From Figure 2, it is evident that the LD decay of Yunling cattle is faster. This outcome is in line with Qiuming Chen et al.’s research, suggesting a potential lack of genetic diversity in the Yunling cattle breed [8].

In 2021, Liu et al. computed the nucleotide diversity, observed heterozygosity, expected heterozygosity and inbreeding coefficient of Chinese indigenous breeds and Western breeds [30], and we used the same methodology and parameters to evaluate the relevant indices of Yunling cattle (Table 1). With the exception of Yunling cattle, all the data in Table 1 are sourced from the research conducted by Liu et al. [30]. The pi value in Yunling cattle (0.000166) is notably lower than that of most Chinese indigenous and Western breeds, as shown in Table 1. Table 1 shows that the $H_{O}$ value of various breeds of cattle ranged from 0.094 to 0.317, with Yunling cattle ranking fourth (0.177) among all other breeds. The value of $H_{E}$ ranged from 0.089 to 0.279, with Yunling cattle ranking third from last (0.256) among all other breeds. It was unexpected that, in the Yunling cattle, the value of $H_{O}$ was significant smaller than that of $H_{E}$. Additionally, in the whole population, the Yunling cattle displayed the highest heterozygosity deficit (($H_{E}$ − $H_{O}$)/$H_{E}$), which may indicate a high degree of inbreeding in the population, as shown in Table 1. The results suggest that Yunling cattle display a relatively low level of genetic diversity and a certain amount of inbreeding within the population.

### 3.2. Population Structure

**Figure 3:**
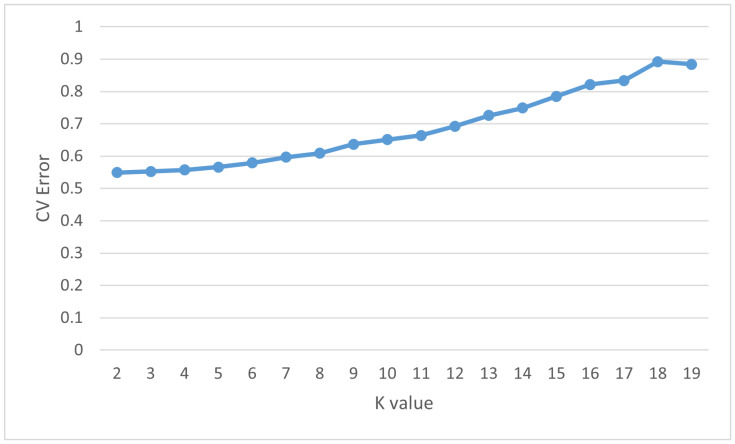
Cross-validation error of admixture analysis (K from 2 to 19). The *x*-axis represents the number of predetermined subpopulations, while the vertical axis shows the cross-validation error obtained from the ADMIXTURE analysis.

**Figure 4:**
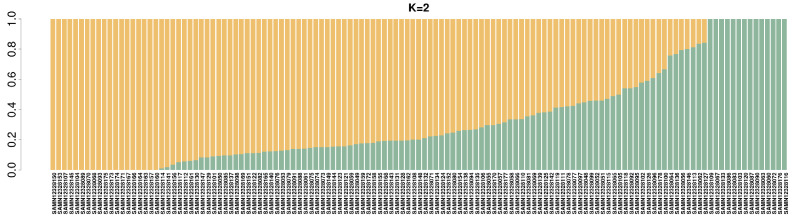
Population structure analysis of the 129 Yunling cattle individuals accomplished from K = 2. The BioSample numbers for the individual references are shown in labels. Each vertical bar represents a sample and the percentage of the colour indicates the probability of that sample being assigned to that subgroup. Orange for subgroup I; green for subgroup II.

Based on the ADMIXTURE analysis, it appears that the population is divided into two subgroups, as indicated by the CV error value, which was at its lowest point with a K value of 2 (Figure 3). When K = 2, the 29 individuals were clustered into a small group, while the rest of the individuals formed a markedly larger group. The result of ADMIXTURE when K = 2 is shown in Figure 4. In Figure 4, every individual is depicted by a slender vertical bar, which is divided into two coloured segments on the *y*-axis (orange = group I; green = group II). The lengths of these segments are proportional to the estimated probability of the group.

**Figure 5:**
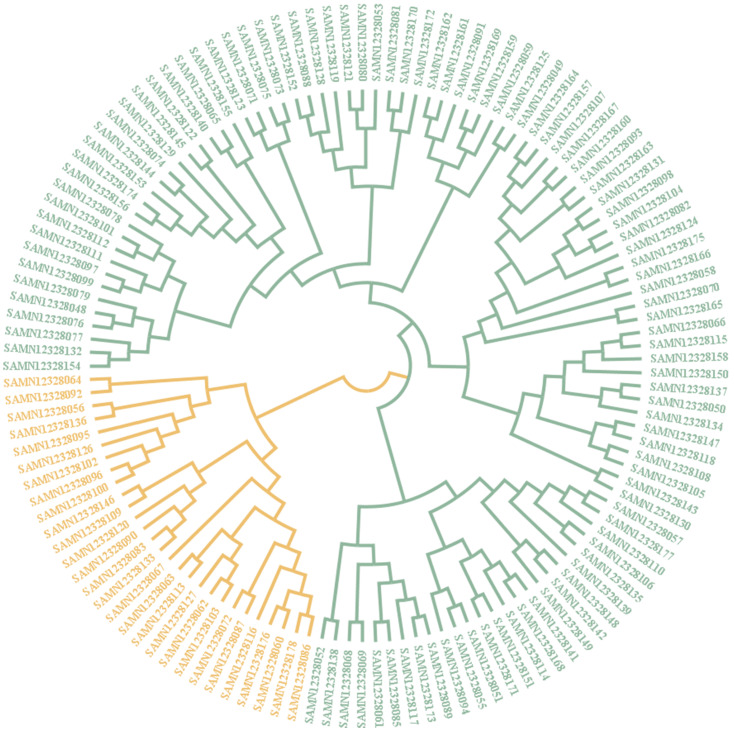
Phylogenetic tree of the relationship among 129 Yunling cattle individuals. The BioSample numbers for the individual references are shown in labels. Orange for subgroup I; green for subgroup II.

The results of the neighbour-joining tree analysis were basically in agreement with the results of the ADMIXTURE analysis, and the 129 individuals were separated into two main groups, named subgroups I and II (Figure 5). However, the classification of individuals is not entirely consistent with ADMIXTURE. Subgroup I contains 28 individuals, while subgroup II consists of the remaining 101 individuals. The sample numbers of the two subgroups are shown in Supplementary Table S3. The genetic relationship among Yunling cattle was determined using a neighbour-joining dendrogram, which was constructed based on the genetic distance between individual animals.

We performed PCA on the genetic distance matrix between individuals and extracted the first three principal components. OPTICS clustering was performed on the three principal components. As a result, Yunling cattle could be classified into two subgroups, which is concordant with the results of NJ-tree analysis and admixture analysis. Not surprisingly, PCA’s classification of the Yunling cattle group is not much different from the first two methods. The group I divided by PCA contains 40 individuals, and the remaining 89 individuals form subgroup II.

**Figure 6:**
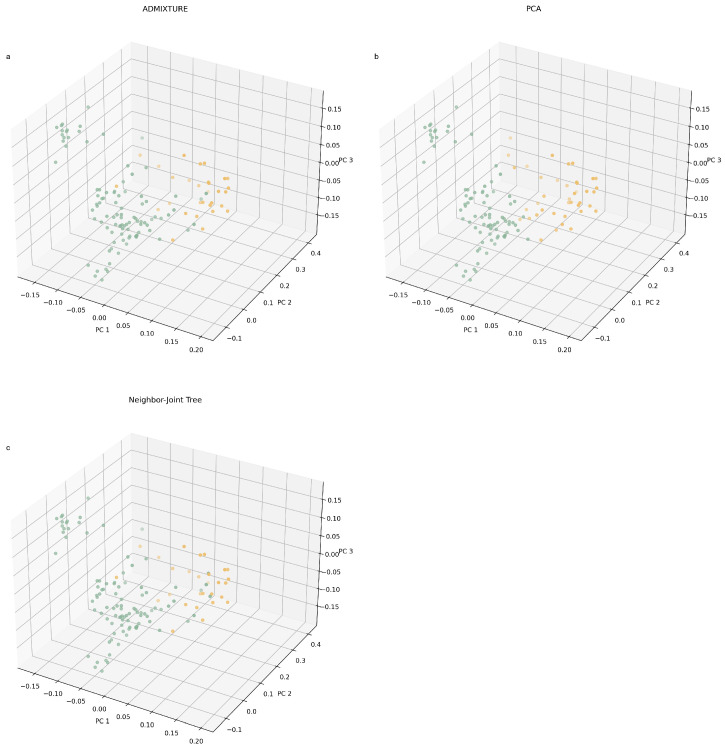
The result of classification of 129 Yunling cattle individuals based on three different methods (orange = group I; green = group II). (**a**) The result of ADMIXTURE grouping. (**b**) The result of neighbour-joining tree grouping. (**c**) The result of PCA clustering grouping.

To facilitate an intuitive comparison of the distinctions among the three grouping methods in classifying individuals, we constructed a three-dimensional scatterplot separately based on the first three principal components to visualise the associations between individuals. A three-dimensional plot is generated using the respective value of every sample across the first (PC1), second (PC2) and third (PC3) principal components. On the basis of the above three figures, we coloured the individuals in the figure according to the clustering results and obtained three figures corresponding to ADMIXTURE (Figure 6a), NJ-tree (Figure 6b) and PCA (Figure 6c), respectively. The clustering results of the three methods are shown in Supplementary Table S3.

### 3.3. Inbreeding Analysis and Subgroup Analysis

To have a better understanding of the genetic status of Yunling cattle, we calculated the homozygosity and heterozygosity of the two subpopulations of Yunling cattle, respectively (Table 2). From the data presented in Table 2, it can be observed that the heterozygosity in both subgroup I and subgroup II is lower than the expected heterozygosity. This indicates a certain level of inbreeding within each of the two subgroups, which is consistent with the analysis of the entire group.

To evaluate the level of inbreeding within the Yunling cattle population, an estimation of the excess of homozygosity inbreeding coefficient ($F_{HOM}$) was computed. The results indicate that the mean value of $F_{HOM}$ in Yunling cattle (0.251) was at the intermediate level (Table 2), while the average values of $F_{HOM}$ in subgroup I and II were similar to those of the Yunling cattle group at a higher level. All the above evidence points to the occurrence of inbreeding events in Yunling cattle.

In order to analyse the breeding situation of Yunling cattle populations, we calculate fixation indexes. Among these measures, $F_{IS}$ is used to indicate the relative degree of inbreeding of individuals within a breed, with higher values indicating a greater degree of inbreeding [31]. The $F_{IS}$ score of Yunling cattle is 0.309, which indicates a noteworthy level of inbreeding in Yunling cattle. $F_{ST}$ quantifies the extent of genetic difference among breeds, with higher $F_{ST}$ values representing greater genetic distances [31]. We utilised $F_{ST}$ to estimate the differentiation among Yunling cattle population. The $F_{ST}$ value of the Yunling cattle population is 0.0274, which indicates a certain degree of differentiation between subgroup I and subgroup II. Meanwhile, this conclusion is consistently in line with the results of ADMIXTURE analysis, phylogenetic tree analysis and PCA analysis.

## 4. Discussion

Livestock and poultry germplasm resources are strategic resources that guarantee the provision of national livestock products and serve as the fundamental basis for the rejuvenation of the seed industry. The objective of conservation work is to maintain the genetic diversity of the population, mitigate the adverse effects of genetic drift and gene deletion, secure the thriving development and adaptability of the population to its environment and stimulate the enhancement and innovation of livestock and poultry breeds [32]. Scientific evaluation of conservation effects can ascertain the existence of genetic risks in current populations and identify necessary interventions to alleviate them. The Yunling cattle breed is one of an excellent local breed in China and holds a significant position in the development of my country’s livestock industry. However, the present comprehensive genome-wide genetic diversity and population structure are insufficient. Therefore, this research has assembled and annotated the genome of Yunling cattle by using whole-genome resequencing technology to examine 129 Yunling cattle specimens from the core breeding facility of the Yunnan Academy of Grassland and Animal Science. The study investigated genetic diversity, population structure and inbreeding status to provide direction for Yunling cattle breeding. The research findings serve as a scientific basis for the formulation of a Yunling cattle breeding protection plan.

In this study, we observed that, compared to other breeds, Yunling cattle exhibited decreased levels of nucleotide diversity. The decreased level of nucleotide diversity is potentially due to inbreeding or selective breeding during the formation of Yunling cattle in the last 30 years. This is consistent with previously reported findings on diversity, global and local pedigree components [8]. Population structure analysis helps us to determine the relationships and interactions between individuals in a population. In our study, we identified that the Yunling cattle population was differentiated into two subgroups.

Mating among closely related individuals can result in several negative consequences, such as diminished genetic diversity within the population, amplified danger of disease in offspring, heightened impact of genetic drift and inbreeding depression. In this study, we confirm that there has been inbreeding in the Yunling cattle population, which has impacted both subgroups. This is one of the reasons why the genetic diversity in Yunling cattle is at a low level and the species advantage is degraded (decreased meat quality). In this context, the results of our study on the population structure and genetic diversity in Yunling cattle will be an important basis for the development of a scientific breeding programme for Yunling cattle and better conservation of the genetic diversity in this breed.

In summary, we have analysed the whole-genome sequence data of 129 Yunling cattle, relying on the assembled file and annotated file of the Yunling cattle genome in this study. The outcomes of the genetic structure and diversity analyses indicate that the Yunling cattle breed should be regarded as a precious genetic resource. There is an urgent need to optimise the breeding system to maintain sufficient genetic diversity and prevent the onset of inbreeding depression.

## Figures and Tables

**Table 1:** Genetic diversity among the 26 cattle breeds.

Breed	Category	Area	Size	Pi	$H_{O}$	$H_{E}$	$F_{\mathrm{HOM}}$	(($H_{E}-H_{O}$)/$H_{E}$)
Yunling	*B. indicus*	China	129	0.000166	0.177	0.256	0.251	0.309
Bohai Black	Hybrid	China	40	0.000334	0.191	0.259	0.313	0.263
Dabieshan	Hybrid	China	4	0.000404	0.252	0.243	0.083	−0.037
Jiaxian Red	Hybrid	China	5	0.000389	0.252	0.247	0.086	−0.02
Lingnan	Hybrid	China	8	0.000392	0.235	0.255	0.145	0.078
Luxi	Hybrid	China	7	0.000371	0.282	0.236	0.026	−0.195
Nanyang	Hybrid	China	7	0.000433	0.317	0.279	0.154	−0.136
Zaobei	Hybrid	China	5	0.0004	0.262	0.247	0.046	−0.06
Guangfeng	*Bos indicus*	China	4	0.000336	0.203	0.188	0.26	−0.08
Ji’an	*B. indicus*	China	4	0.000325	0.195	0.176	0.29	−0.108
Jinjiang	*B. indicus*	China	4	0.000368	0.241	0.217	0.123	−0.111
Leiqiong	*B. indicus*	China	3	0.000321	0.176	0.153	0.358	−0.15
Wannan	*B. indicus*	China	6	0.000302	0.193	0.174	0.298	−0.109
Kazakh	*Bos taurus*	China	11	0.000356	0.232	0.236	0.154	0.017
Mongolian	*B. taurus*	China	9	0.000369	0.243	0.241	0.115	−0.008
Xuanhan	*B. taurus*	China	5	0.000395	0.262	0.246	0.044	−0.065
Charolais	*B. taurus*	Europe	10	0.00034	0.22	0.221	0.199	0.005
Jersey	*B. taurus*	Europe	20	0.000281	0.186	0.187	0.326	0.005
Limousin	*B. taurus*	Europe	10	0.000327	0.221	0.213	0.197	−0.038
Simmental	*B. taurus*	Europe	30	0.000327	0.22	0.22	0.199	0
Hanwoo	*B. taurus*	South Korea	11	0.000328	0.22	0.214	0.199	−0.028
Mishima-Ushi	*B. taurus*	Japan	8	0.0002	0.094	0.089	0.657	−0.056
Angus	*B. taurus*	Europe	33	0.000309	0.208	0.208	0.242	0
Hereford	*B. taurus*	Europe	21	0.000288	0.191	0.194	0.309	0.015
Holstein	*B. taurus*	Europe	20	0.000301	0.2	0.201	0.273	0.005
Brahman	*B. indicus*	India	5	0.000268	0.143	0.173	0.47	0.173

pi, average nucleotide diversity; $H_{E}$, expected heterozygosity; $H_{O}$, observed heterozygosity; $F_{HOM}$, excess of homozygosity inbreeding coefficient.

**Table 2:** Inbreeding indicators of Yunling cattle population and its subpopulations.

Group	$H_{O}$	$H_{E}$	$F_{\mathrm{HOM}}$	(($H_{E}$ − $H_{O}$)/$H_{E}$)
Subgroup I	0.175	0.254	0.23	0.311
Subgroup II	0.177	0.256	0.246	0.309
Yunling cattle	0.177	0.256	0.251	0.309

## Data Availability

The reference genome data are available from GenBank with the Bioproject accession number PRJNA978937.

## Supplementary Materials

The following supporting information can be downloaded at: https://www.mdpi.com/article/10.3390/genes14122141/s1, Figure S1: Density plots for filtered SNP quality; Figure S2: Distribution of SNPs on chromosomes; Table S1: Overview of sample information and sequencing statistics; Table S2: Mapping rate of Yunling cattle sequencing data aligned to the Yunling cattle reference genome; Table S3: Results of population structure differentiation using three methods: admixture, NJ tree and PCA.

## References

[1] Qu K.X., Huang B.Z., Yang G.R., He Z.X., Zhang Y.P., Zan L.S. (2012). Genetic diversity analysis of BMY cattle based on microsatellite DNA markers. Russ. J. Genet..

[2] Xiaoting X., Kaixing Q., Fangyu L., Peng J., Qiuming C., Ningbo C., Jicai Z., Hong C., Bizhi H., Chuzhao L. (2019). Abundant Genetic Diversity of Yunling Cattle Based on Mitochondrial Genome. Animals.

[3] Yan B. (2019). Population Genomics Research on Chaidamu Yellow Cattle. Master’s Thesis.

[4] Bravo S., Larama G., Quiñones J., Paz E., Rodero E., Sepúlveda N. (2019). Genetic diversity and phylogenetic relationship among araucana creole sheep and Spanish sheep breeds. Small Rumin. Res..

[5] Xianbo J., Peng D., Shiyi C., Shaokang Z., Jie W., Songjia L. (2021). Analysis of MC1R, MITF, TYR, TYRP1, and MLPH Genes Polymorphism in Four Rabbit Breeds with Different Coat Colors. Animals.

[6] Qu K., Zhang J., Yang G., He Z., Jin X., Wang A., Yuan X., Huang B., Zan L. (2011). Karyotypic analysis of BMY cattle and Brahman. J. Northwest A & F Univ.-Nat. Sci. Ed..

[7] Xia X., Yao Y., Li C., Zhang F., Qu K., Chen H., Huang B., Lei C. (2019). Genetic diversity of Chinese cattle revealed by Y-SNP and Y-STR markers. Anim. Genet..

[8] Chen Q., Zhan J., Shen J., Qu K., Hanif Q., Liu J., Zhang J., Chen N., Chen H., Huang B. (2020). Whole-genome resequencing reveals diversity, global and local ancestry proportions in yunling cattle. J. Anim. Breed. Genet..

[9] Sambrook J., Russell D.W., Irwin C.A., Janssen K.A. (2001). Molecular Cloning: A Laboratory Manual.

[10] Deorowicz S., Kokot M., Grabowski S., Debudaj-Grabysz A. (2015). KMC 2: Fast and resource-frugal k-mer counting. Bioinformatics.

[11] Vurture G.W., Sedlazeck F.J., Nattestad M., Underwood C.J., Fang H., Gurtowski J., Schatz M.C. (2017). GenomeScope: Fast reference-free genome profiling from short reads. Bioinformatics.

[12] Servant N., Varoquaux N., Lajoie B.R., Viara E., Chen C.J., Vert J.P., Heard E., Dekker J., Barillot E. (2015). HiC-Pro: An optimized and flexible pipeline for Hi-C data processing. Genome Biol..

[13] Langmead B., Salzberg S.L. (2011). Fast gapped-read alignment with Bowtie 2. Nat. Methods.

[14] Burton J.N., Adey A., Patwardhan R.P., Qiu R., Kitzman J.O., Shendure J. (2013). Chromosome-scale scaffolding of de novo genome assemblies based on chromatin interactions. Nat. Biotechnol..

[15] Kaymak I., Luda K.M., Duimstra L.R., Ma E.H., Longo J., Dahabieh M.S., Faubert B., Oswald B.M., Watson M.J., Kitchen-Goosen S.M. (2022). Carbon source availability drives nutrient utilization in CD8+ T cells. Cell Metab..

[16] Zdobnov E.M., Apweiler R. (2001). InterProScan—An integration platform for the signature-recognition methods in InterPro. Bioinformatics.

[17] Lowe T.M., Eddy S.R. (1997). tRNAscan-SE: A Program for Improved Detection of Transfer RNA Genes in Genomic Sequence. Bioinformatics.

[18] Griffiths-Jones S., Moxon S., Marshall M., Khanna A., Eddy S.R., Bateman A. (1997). Rfam: Annotating non-coding RNAs in complete genomes. Bioinformatics.

[19] Lagesen K., Hallin P., Rødland E.A., Stærfeldt H.H., Rognes T., Ussery D.W. (1997). RNAmmer: Consistent and rapid annotation of ribosomal RNA genes. Bioinformatics.

[20] Li H., Durbin R. (2009). Fast and accurate short read alignment with Burrows-Wheeler transform. Bioinformatics.

[21] Nekrutenko A., Taylor J. (2012). Next-generation sequencing data interpretation: Enhancing reproducibility and accessibility. Nat. Rev. Genet..

[22] Wei Z. The Assembled Draft Genome of Yunling Cattle Annotation Results of Repeated Sequences, Gene Structure and Functional Prediction. https://figshare.com/articles/dataset/The_assembled_draft_genome_of_Yunling_cattle_annotation_results_of_repeated_sequences_gene_structure_and_functional_prediction_/23391614/1.

[23] Wang K., Li M., Hakonarson H. (2010). ANNOVAR: Functional annotation of genetic variants from high-throughput sequencing data. Nucleic Acids Res..

[24] Zhang C., Dong S.S., Xu J.Y., He W.M., Yang T.L. (2019). PopLDdecay: A fast and effective tool for linkage disequilibrium decay analysis based on variant call format files. Nucleic Acids Res..

[25] Purcell S., Neale B., Todd-Brown K., Thomas L., Ferreira M.A.R., Bender D., Maller J., Sklar P., de Bakker P.I.W., Daly M.J. (2007). PLINK: A tool set for whole-genome association and population-based linkage analyses. Am. J. Hum. Genet..

[26] Danecek P., Auton A., Abecasis G., Albers C.A., Banks E., DePristo M.A., Handsaker R.E., Lunter G., Marth G.T., Sherry S.T. (2011). The variant call format and VCFtools. Bioinformatics.

[27] Alexander D.H., Lange K. (2011). Enhancements to the ADMIXTURE algorithm for individual ancestry estimation. BMC Bioinform..

[28] Yang J., Lee S.H., Goddard M.E., Visscher P.M. (2011). GCTA: A tool for genome-wide complex trait analysis. Am. J. Hum. Genet..

[29] Kumar S., Stecher G., Li M., Knyaz C., Tamura K. (2018). MEGA X: Molecular Evolutionary Genetics Analysis across Computing Platforms. Mol. Biol. Evol..

[30] Liu Z., Sun H., Lai W., Hu M., Zhang Y., Bai C., Liu J., Ren H., Li F., Yan S. (2022). Genome-wide re-sequencing reveals population structure and genetic diversity of Bohai Black cattle. Anim. Genet..

[31] Strucken E.M., Gebrehiwot N.Z., Swaminathan M., Joshi S., Kalaldeh M.A., Gibson J.P. (2021). Genetic diversity and effective population sizes of thirteen Indian cattle breeds. Genet. Sel. Evol..

[32] Edwards C.E., Tessier B.C., Swift J.F., Bassüner B., Linan A.G., Albrecht M.A., Yatskievych G.A. (2021). Conservation genetics of the threatened plant species Physaria filiformis (Missouri bladderpod) reveals strong genetic structure and a possible cryptic species. PLoS ONE.

