# Supplementary figures and images for: Population Structure and Genetic Diversity of Yunling Cattle Determined by Whole-Genome Resequencing
Source: Genes (Basel). 2023 Nov 27;14(12):2141. doi: 10.3390/genes14122141 (PMC10742670; doi:10.3390/genes14122141)

Figure S1 density plots for filtered SNP quality

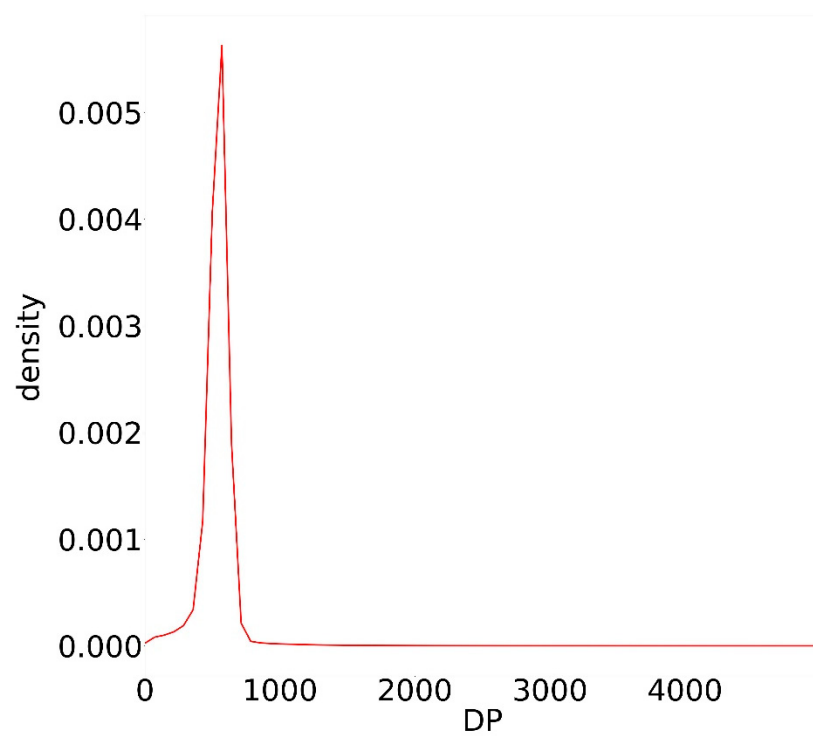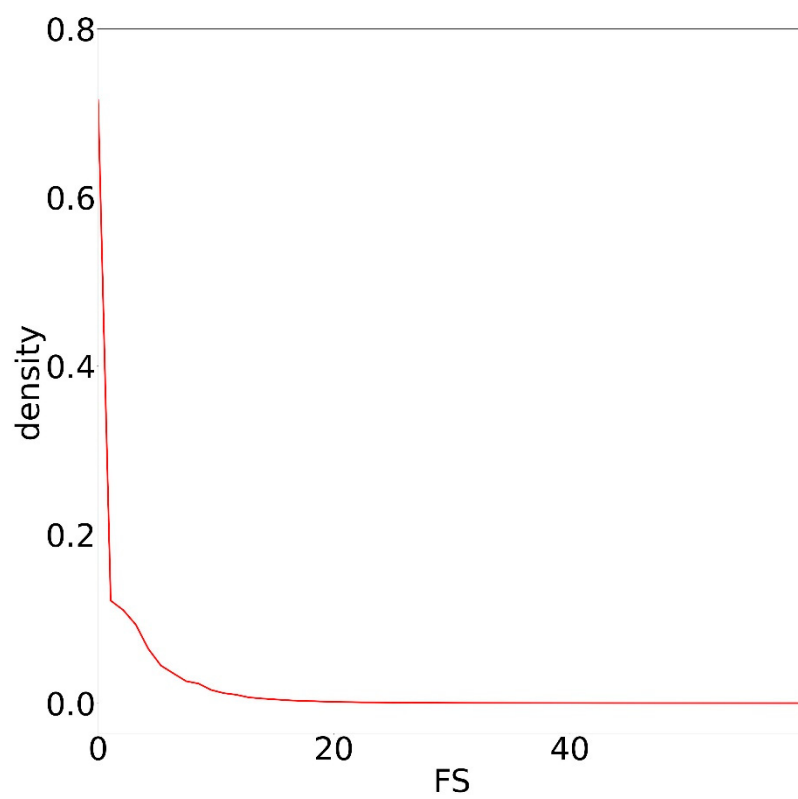

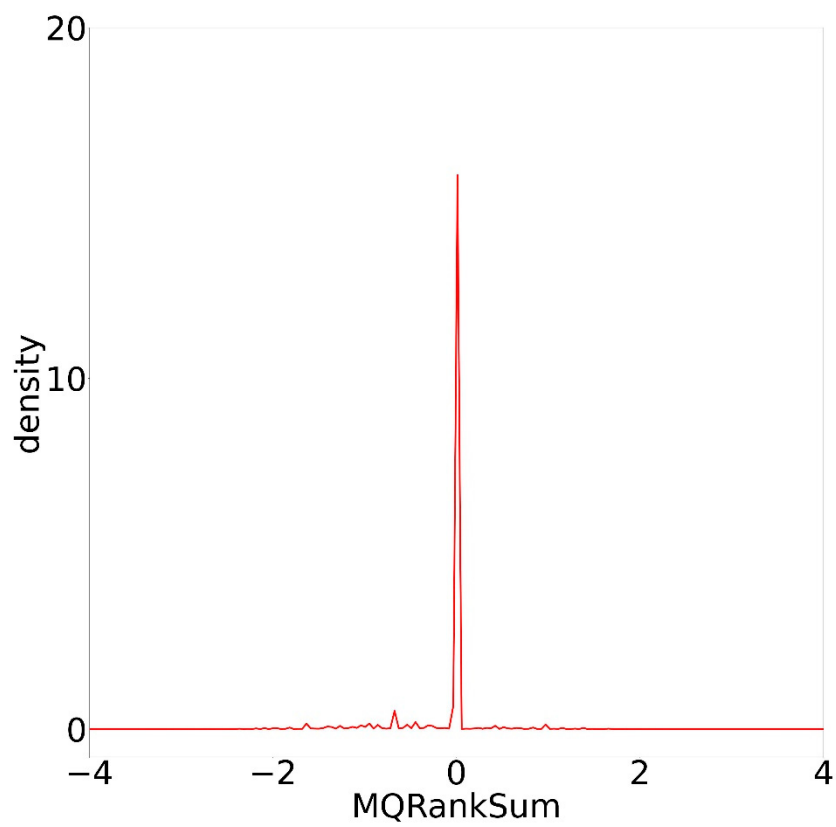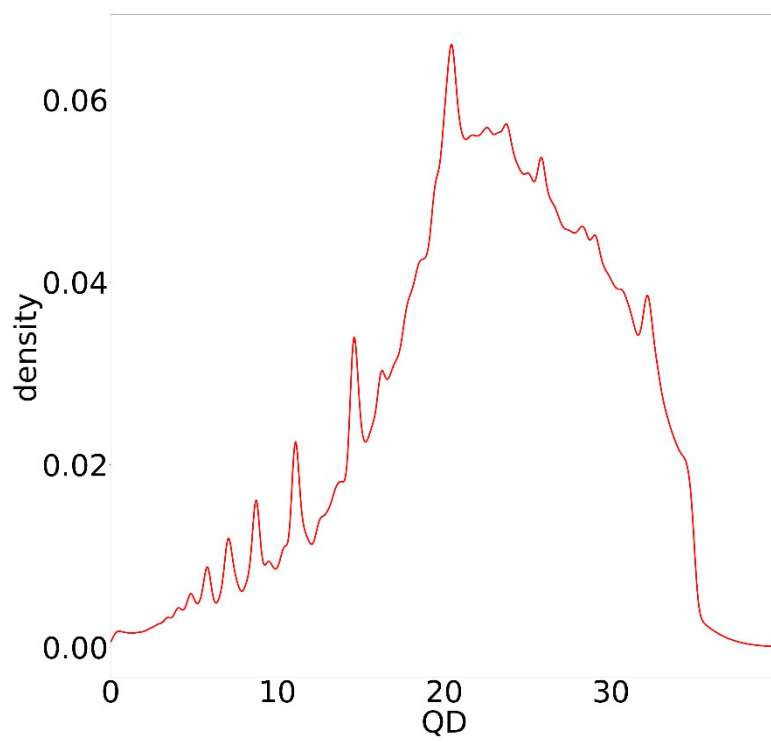

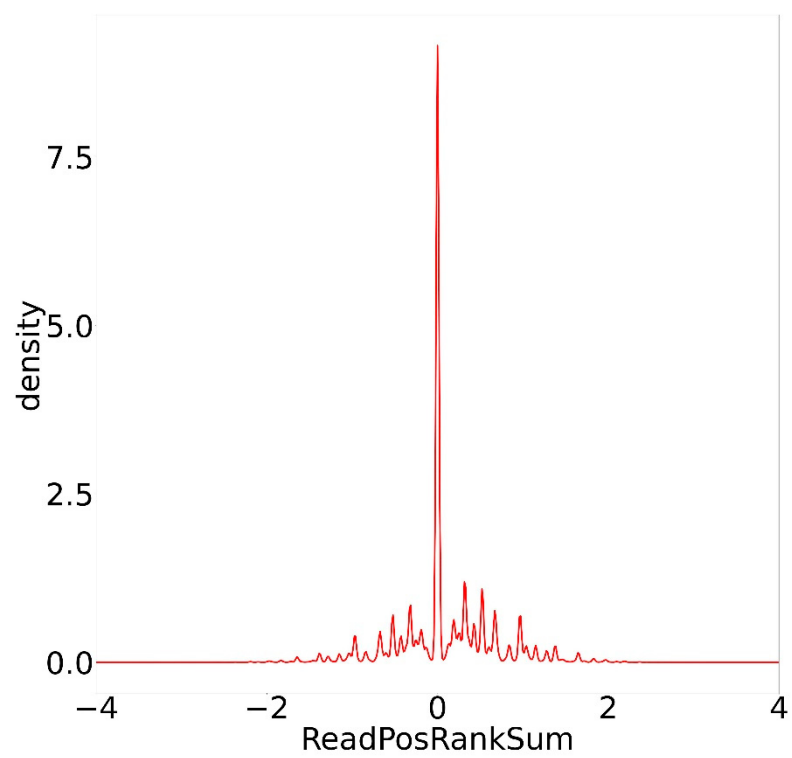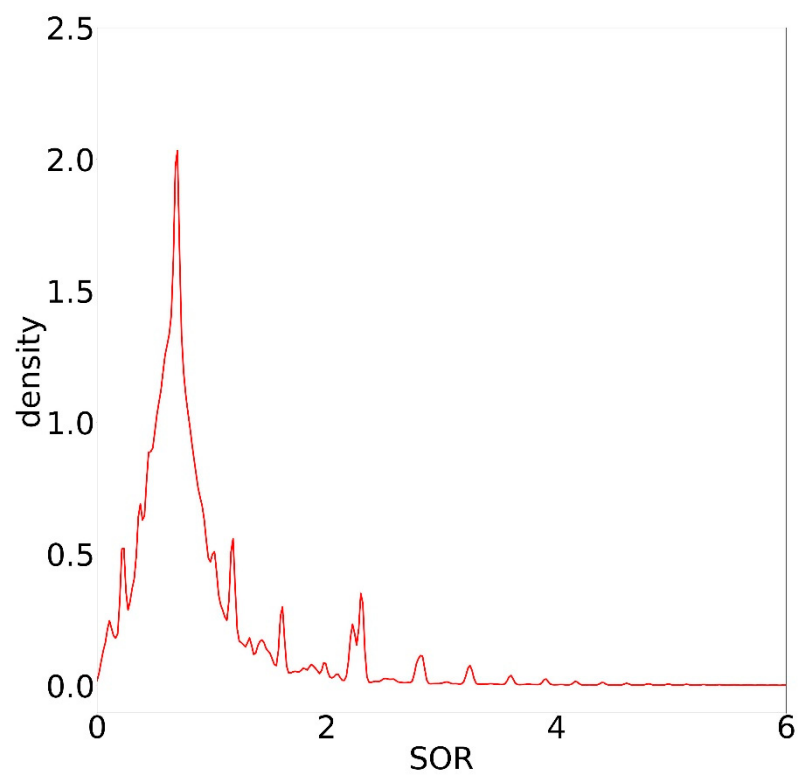

Figure S2 Distribution of SNPs on chromosomes

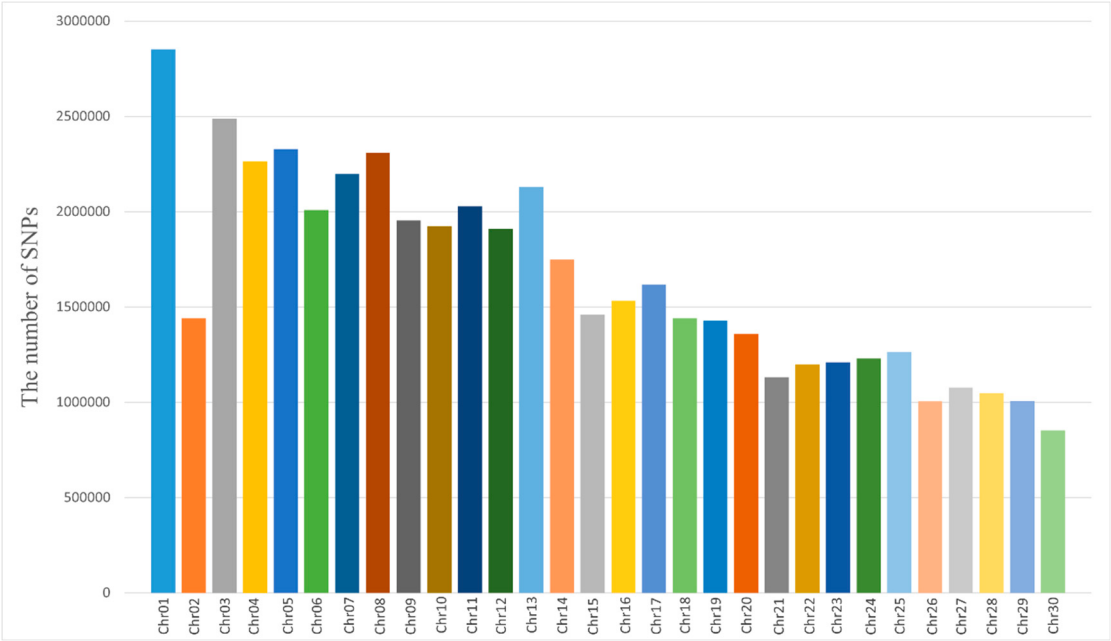

Supplement: Supplementary file 1 [file genes-14-02141-s001.zip › Supply information-Figures.pdf]
